# Pediatric Wilson Disease in Sudan: A Rare Case, Sudan Conflict and Diagnostic Challenges

**DOI:** 10.1002/ccr3.72849

**Published:** 2026-06-04

**Authors:** Amjed Abdu Ali Mohammed, Anaheed Alzubair Mohammed HabeebAllah, Sudad salahaldeen Hassan Mohammed, Aprar Kamal Mohammed Ali Almamoon, Mehad Mortada BadrAlden Ahmed, Arafa Mubarak Abotalib Aref, Fatima Bashir Omer Bashir, Tarig Mohamed Nourallah Altegani, Ahmed Alshafei Elmahi Ahmed

**Affiliations:** ^1^ Faculty of Medicince Khartoum University Khartoum Sudan; ^2^ Faculty of Medicine and Health Science Nile Valley University Atbara Sudan; ^3^ Faculty of Medicine University of Gezira Wad Madani SD Sudan; ^4^ Faculty of Medicine and Health Science Omdurman Islamic University Khartoum Sudan; ^5^ Faculty of Medicince The National Ribat University Khartoum Sudan

**Keywords:** case report, Kayser‐Fleischer rings, neuropsychiatric symptoms, pediatrics, Sudan, Wilson disease

## Abstract

Wilson disease is rarely reported among African children. Wilson disease is due to a mutation in ATP7B on an autosomal recessive pattern, which causes defective copper excretion and copper accumulation in tissues such as liver and brain. To the best of our knowledge, this is the second reported case of Wilson disease from Sudan, and it highlights the challenges of diagnosis and management in low‐resource settings. Our report strengthens the need for awareness and accountability of rare diseases in public health systems, especially when access to diagnostics and special therapies is limited. A 13‐year‐old male of Sudanese descent with consanguineous parents presented with progressive generalized edema, jaundice, and ascites. On physical examination, Kayser‐Fleischer rings were observed. Laboratory tests revealed hypoalbuminemia, thrombocytopenia, and a family history of sibling deaths. Additionally, there was a difference in neuropsychiatric status. MRI of the brain demonstrated hyperintensities in the basal ganglia, known as the “giant panda sign.” A diagnosis of Wilson disease was made, with a Leipzig score of 7/10, based on low serum copper, hemolytic anemia, and the family history. Initial therapy with D‐penicillamine and then zinc was administered, but it failed to prevent neurological decline. This deterioration occurred between visits. Moreover, the only alternate therapy, trientine, in all its forms, was not available at the time. Wilson disease, thought rare, occurs in African children but remains underdiagnosed due to resource constraints. This case highlights diagnostic challenges, the critical role of clinical suspicion, and urgent need for accessible diagnostic and affordable therapies in low‐resource settings to improve outcome.

## Key Clinical Message

Wilson disease should be suspected in African children with unexplained hepatic or neuropsychiatric symptoms. In conflict‐affected low‐resource settings, use clinical clues (Kayser‐Fleischer rings, MRI) and the Leipzig score for early diagnosis. Second‐line therapies may be unavailable, and war disrupts first‐line treatment. Diagnosis alone is insufficient without sustained therapeutic access.

**Abbreviations**
ALP: Alkaline Phosphatase
ALT: Alanine Aminotransferase
ANA: Antinuclear Antibody
ASMA: Anti‐Smooth Muscle Antibody
AST: Aspartate Aminotransferase
ATP7B: ATPase Copper Transporting Beta
C3/C4: Complement Components 3 and 4
CNS: Central Nervous System
DWI/ADC: Diffusion‐Weighted Imaging/Apparent Diffusion Coefficient
EASL: European Association for the Study of the Liver
Hb: Hemoglobin
INR: International Normalized Ratio
KF: Kayser‐Fleischer
LKM: Liver‐Kidney Microsomal (antibodies)
MCV: Mean Corpuscular Volume
MRI: Magnetic Resonance Imaging
T2/FLAIR: T2‐Weighted/Fluid‐Attenuated Inversion Recovery
TDS: ter die sumendum (three times daily)
WD: Wilson Disease

## 1 Introduction

Wilson disease, or hepatolenticular degeneration, is a rare genetic disorder of autosomal recessive inheritance caused by a mutation in the ATP7B gene on chromosome 13 (13q14.3) [1]. This mutation results in an impairment in copper metabolism due to dysfunction of the hepatic copper‐transporting P‐ATPase. Therefore, biliary copper excretion is reduced, resulting in pathological copper accumulation in numerous organs, especially the liver and brain. This excess copper is toxic, creates a state of oxidative stress, and ultimately leads to injury to a wide variety of organ systems [1]. The clinical manifestations of Wilson disease stem from organ‐specific susceptibility based on duration of exposure to copper, copper load and organ regenerative ability. Hepatic manifestations typically come first due to initial copper deposition occurring in the liver; although the hepatic regenerative capacity can initially help compensate for the damage sustained by the organ. The blood–brain barrier limits copper accumulation in the CNS initially, thus neurotoxic effects appear to have a delayed onset. However, after the barrier is compromised, the CNS has an enormous vulnerability to copper toxicity because of limited regenerative ability and sensitivity to injury caused by heavy metals [2]. Clinically, Wilson disease (WD) commonly manifests with hepatic dysfunction, which includes features such as hepatomegaly and acute liver failure. In addition, copper accumulation in the basal ganglia leads to neurological symptoms, including parkinsonian features, as well as psychiatric manifestations such as depression and psychosis. If left untreated, WD can result in irreversible organ damage and severe disability [3]. WD has a global prevalence of approximately 1 in 30,000 individuals, with a relatively uniform distribution across ethnic groups. However, certain consanguineous populations exhibit a higher gene frequency, likely due to a founder effect, as suggested by haplotype studies. Globally, the carrier rate is estimated at around 1 in 100 individuals, with an annual incidence ranging from 15 to 25 cases per million [4]. The diagnostic difficulties experienced in Wilson disease (WD) is resolved by Leipzig criteria that has incorporated the findings of the 8th International Conference on Wilson Disease and Menkes Disease in Leipzig (2001). This validated scoring system allows you to take into account clinical findings (neuropsychiatric symptoms, Kayser‐Fleischer rings), amounts of biochemical measures (low serum ceruloplasmin, high urinary copper), histopathological findings (hepatic copper quantifications), and identify molecular genetic studies (ATP7B mutations) contributing towards clinical diagnostic decision making in the context of clinical features. Patients scored with a total greater than 4 can be diagnosed with WD allowing for clinical management still while if was clinically ambiguous, or early on in disease trajectory [1]. WD can still be treated when diagnosed early. Treatment options include chelation agents such as D‐penicillamine and trientine, and zinc salts through different mechanisms, which eliminate excess copper. If WD treatment is unsuccessful, liver transplantation can be considered. Further, innovative treatment options such as gene therapy are currently being studied with animal models [3].

Our case report describes a 13‐year‐old boy presented with mild portal hypertension and neuropsychiatric symptoms; despite initiation of appropriate treatment, disease progression occurred, exacerbated by storage of essential medication due to the ongoing war in Sudan.

This case highlights the critical impact of health disparities on timely diagnosis and management, emphasizing the need for improving access to essential therapies and alternative treatment strategies for refractory cases. Finally, this case illustrates the complications that can arise in diagnosing and treating Wilson disease in resource‐limited systems when the prognosis is ultimately death.

## 2 Case Presentation

A 13‐year‐old male presented to the emergency department of Atbara Teaching Hospital with a 3‐month history of progressive generalized body swelling. The swelling initially appeared in the periorbital region, then extended to the abdomen, and subsequently involved the lower limbs, without diurnal variation. Concurrently, the patient reported intermittent jaundice over the same period. The jaundice presented in a spontaneous on‐and‐off pattern, not associated with pruritus, altered urine or stool color, or sleep disturbances. The patient was initially seen at a rural primary health center, where doctors suspected nephrotic syndrome as the cause of the generalized swelling. However, all investigations ruled out this possibility. Otherwise, the patient was relatively well and had no other complaints.

### 2.1 Past Medical and Family History

His medical history was significant for consanguinity (second‐degree parental relationship) and three siblings who died before age 20 from undiagnosed illnesses. The observed pattern of early mortality within the family raises concerns regarding a potentially fatal hereditary condition, which requires further investigations to determine its origin.

### 2.2 Physical Exam

**FIGURE 1:**
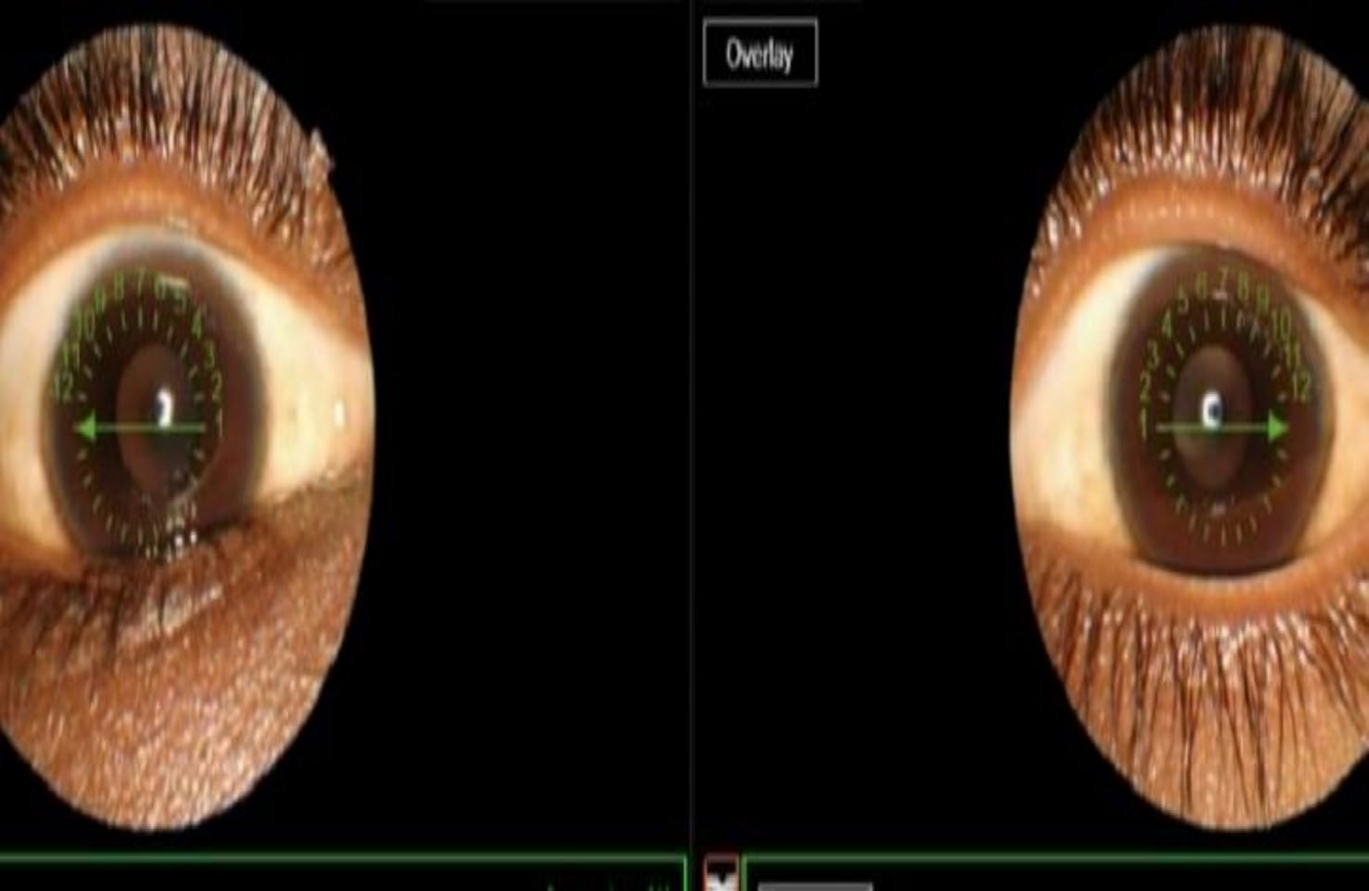
Ptient's bilateral Kayser‐Fleischer rings.

General: Severe pallor, mild jaundice, periorbital edema, and bilateral Kayser‐Fleischer rings (confirmed via slit‐lamp microscopy) (Figure 1).

Abdomen: Massive ascites (positive fluid thrill, shifting dullness); no hepatosplenomegaly.

During admission, he developed agitation, cognitive decline, anhedonia, visual/auditory hallucinations, dysarthria, dysphagia, spasticity, and generalized tonic–clonic seizures.

### 2.3 Workup

**Copper metabolism:** Serum copper 57 μg/dL (reference: 80–190 μg/dL), ceruloplasmin and 24‐h urinary copper unavailable due to resource constraints. **Hepatic:** Elevated AST (75 U/L), ALT (52 U/L), ALP (330 U/L), hypoalbuminemia (1.4 g/dL), normal bilirubin.

**Hematologic:** Normocytic anemia (Hb 9.8 g/dL, MCV 80.7 fL), thrombocytopenia (110 × 10^3^/μL), normal reticulocytes, negative Coombs test.

**Immunologic:** Low C3/C4; negative ANA, ASMA, LKM antibodies.

**Infectious:** Negative hepatitis/hepatic schistosomiasis serology.

**Imaging:** Abdominal ultrasound showed cirrhosis, portal hypertension, and moderate ascites.

**FIGURE 2:**
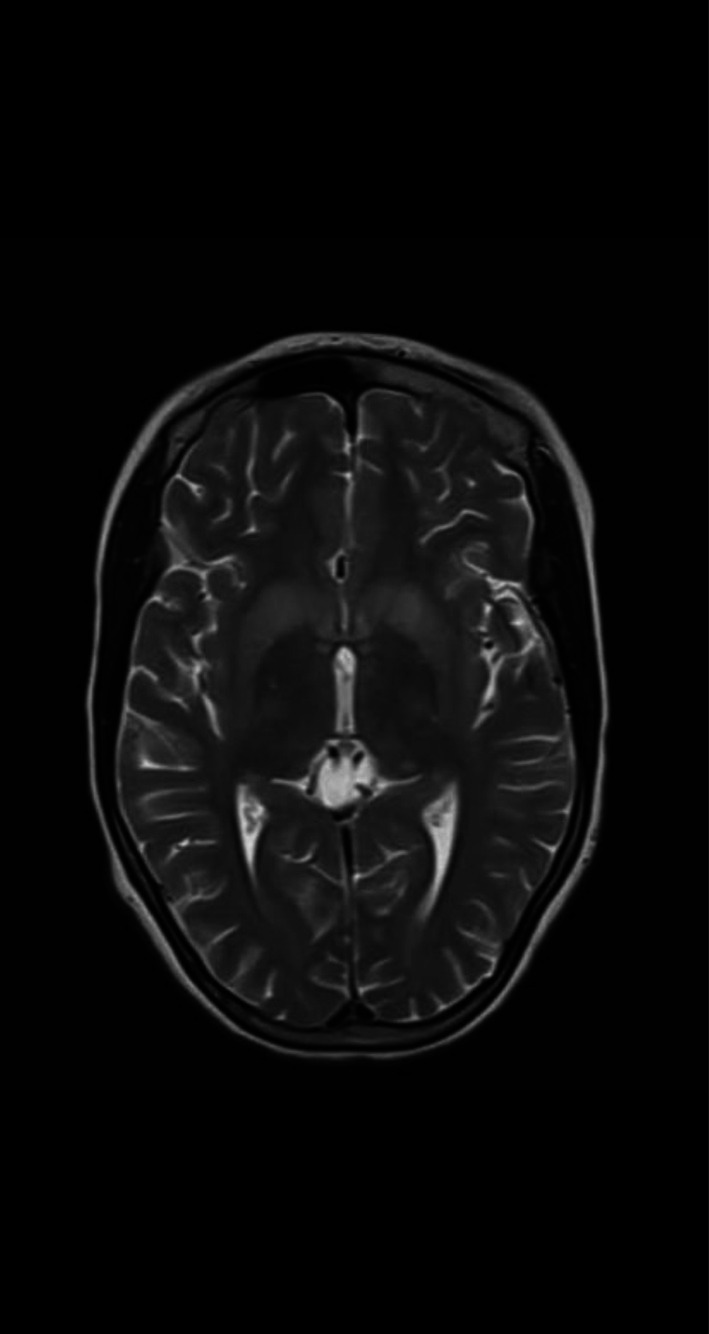
Axial T2 weighted MR image shows hyperintensity in the putamina, caudate nuclei, and ventrolateral thalami (which is the most common abnormality).

**FIGURE 3:**
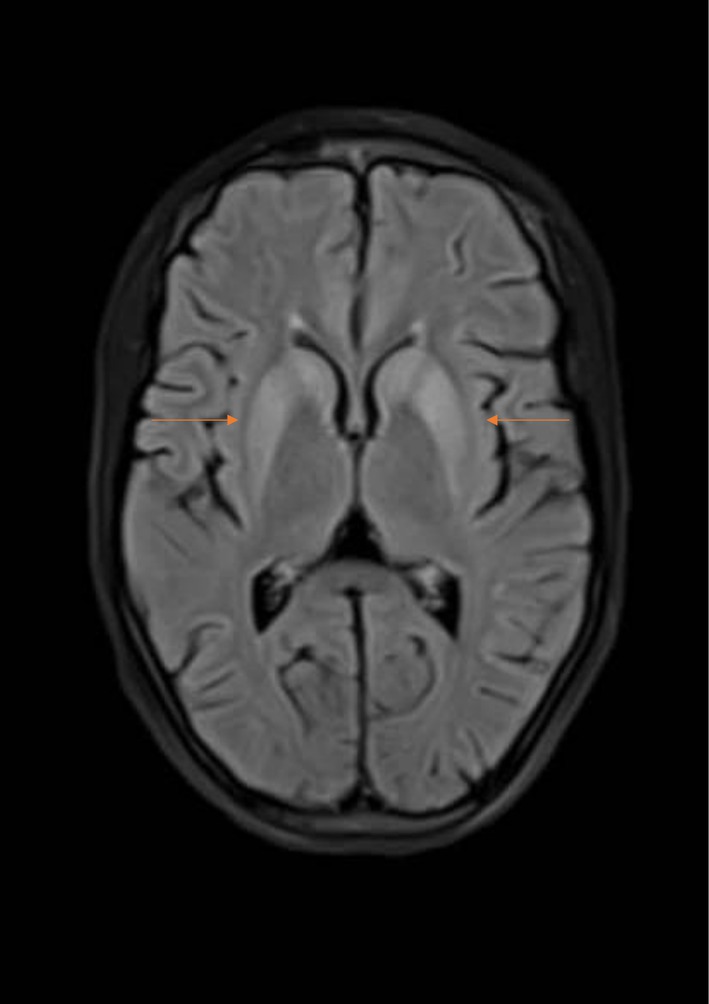
Flair.

**FIGURE 4:**
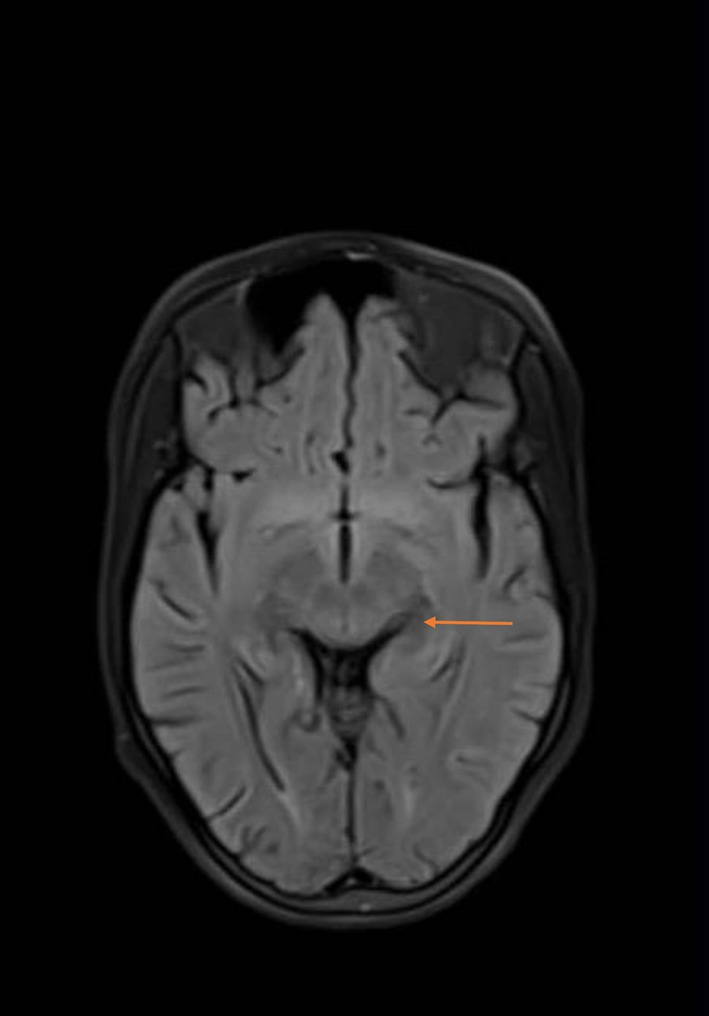
Diffusion restriction (which is rarely seen early in the course of the disease).

**FIGURE 5:**
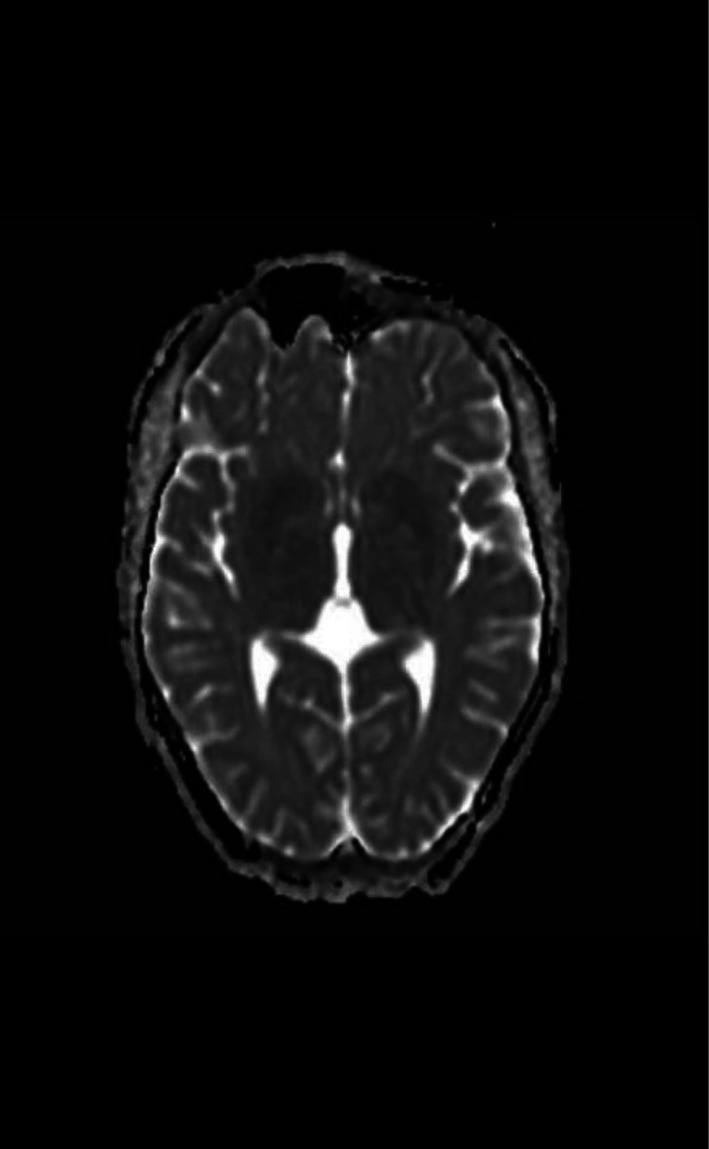
Diffusion restriction (which is rarely seen early in the course of the disease).

**Brain MRI:** abnormal (T2& FLAIR)(Figures 2 and 3) hyperintensity in the putamina, caudate neculei and venterolateral thalami (which is the most common abnormality), with DWI/ADC (Figures 4 and 5): diffusion restriction (which is rarely be seen early in the course of the disease).

### 2.4 Diagnosis

The diagnosis was made using the Leipzig diagnostic scoring system, a validated instrument supported by the European Association for the Study of the Liver (EASL) consensus guidelines from 2001 [5]. The Leipzig scoring system has strong diagnostic validity for Wilson disease (WD), with a sensitivity and specificity of 93%–98% and 92%–96%, respectively.

It has strong discriminatory power distinguishing WD from other hepatic or neurologic disorders with overlapping clinical signs and symptoms, providing consistently reliable diagnostic accuracy in clinically precarious circumstances. (Table 1).

**TABLE 1:** Scoring systems developed at the 8th International Meeting on Wilson's disease in Leipzig 2001.

Typical clinical symptoms and signs		
Evaluation	Score	Our patient acore
Kayser‐Ficher ring		
Present	2	2
Absent	0	
Neurological symptoms		
Severe	2	2
Mild	1	
Absent	0	
Serum ceruloplasmin		
Normal (< 0.2 g/L)	0	_
0.1–0.2 g/L	1	
< 0.1 g/L	2	
Coombs negative hemolytic anemia		
Present	1	1
Absent	0	
Total score		
Diagnosis established	4	5
Diagnosis possible	3	
Diagnosis unlikely	2 or less	

The Leipzig scoring system allows for timely diagnosis of Wilson disease (WD); however, prediction of prognosis is based on validated scoring systems such as the Nazer score, which predicts mortality risk in acute hepatic presentations of WD using serum biochemical markers such as serum bilirubin, INR, and AST. Poor prognostic factors include delayed start to treatment, severe neurological deficits at diagnosis, or lack of compliance with decoppering treatment. Hepatic failure and a Nazer score of more than 11 will require urgent liver transplantation. Starting treatment in presymptomatic or mild presentation cases is associated with good long‐term outcome.

In contrast, poor prognostic factors include delayed treatment initiation, severe neurological deficit at the time of diagnosis, and nonadherence to decoppering therapy; notably, hepatic failure with a prognostic index of Wilson disease > 11 necessitates urgent liver transplantation, whereas early intervention in presymptomatic or mild cases correlates with favorable long‐term outcomes. However, treatment interruptions exemplified in this case by war‐related medication shortages significantly elevate risks of irreversible hepatic or neurological damage, highlighting the critical interplay between diagnostic accuracy, prognostic assessment, and sustained therapeutic access (Table 2) [6].

**TABLE 2:** Prognostic index in Wilson disease.

Parameter	1	2	3	4	Our patient results
Serum bilirubin (mmol/L)	100–150	151–200	201–300	< 300	2
AST (U/L)	100–150	151–300	301–304	< 400	0
INR	1.3–1.6	1.7–1.9	2.0–2.4	< 2.4	1
WBC	6.8–8.3	8.4–10.3	10.4–15.3	< 15.3	0
Albumin (g/L)	34–44	25–33	21–24	> 21	4
Total score					7

## 3 Management

The initial treatment course consisted of D‐penicillamine (starting at 5 mg/kg once a day and increased to 20 mg/kg once a day over the following weeks), zinc sulfate (25 mg tds), pyridoxine (20 mg once a day), lactulose, and albumin. For psychiatric management, haloperidol (3 mg once a day) was used but this was changed to olanzapine (5 mg) due to worsening agitation. Neurological deterioration had occurred by week 3, which was manifested by severe spasticity, dysphagia, decerebrate posturing, and status epilepticus. There was very little response to phenytoin and baclofen. Unfortunately, the second‐line chelator trientine was not available.

## 4 Discharge

The patient's condition rapidly deteriorated, and it became evident that he could no longer tolerate D‐penicillamine. Trientine, the second‐line copper chelating agent, was unavailable. Therefore, we attempted to continue D‐penicillamine at a reduced dose; however, no clinical improvement was observed. To assess the need for urgent liver transplantation, we applied the prognostic index for fulminant Wilsonian hepatitis, which demonstrates high sensitivity in predicting mortality in the absence of transplantation. Our patient scored 7, and urgent liver transplantation was indicated. Currently, we are screening the patient's family members for Wilson disease to enable early diagnosis and management of this fatal but treatable hereditary disorder. In addition, arrangements are being made to facilitate liver transplantation for the patient in India.

## 5 Discussion

Wilson disease (WD) is an autosomal recessive disorder of copper metabolism and is underreported in Sudan where only a few individual cases are documented. The reporting of rare case reports indicates an incomplete epidemiological and clinical record for Sudanese which necessitates more data to improve our understanding of prevalence, diagnostic dilemmas, and treatment outcomes. There may also be genetic and environmental features specific to this region that could affect the presentation and course of disease, warranting local studies [1].

Wilson's disease (WD) arises because of a mutation in the ATP7B gene. This mutation results in a failure to excrete copper from hepatocytes, with copper then being stored in the liver until the storage capacity is exceeded. Once this capacity is exceeded, copper spills into the systemic circulation, and deposits into extra‐hepatic tissues including the brain and Descemet's membrane of the cornea [7, 8], Consistent with large clinical review and study conducted in India [7, 8], our patient presented with hepatic symptoms (jaundice, hepatomegaly) in early adolescence, Although hepatic manifestation of Wilson's disease (WD) classically appear in the first decade of life [7, 8], our case highlights its phenotypic variability, with onset extending beyond this typical window. This aligns with reports of WD's broad age range at presentation, spanning childhood to adulthood [1], underscoring the need to consider WD even in adolescents with hepatic dysfunction. Despite early diagnosis and prompt initiation of treatment, our patient developed neurological and psychiatric complications. This progression emphasizes the unpredictable natural history of WD, which are more commonly reported in later adulthood [9, 10]. Such outcomes reinforce the necessity for vigilant long‐term monitoring and multidisciplinary management in WD, irrespective of early treatment. Clinicians must maintain a high index of suspicion for evolving complications across pediatric and adolescent populations, even in cases deviating from classical age‐related phenotypes, this delay in neurological onset relates to the ability of the liver to deal with excess copper, making the symptom pre‐3 years old rare [1, 11]. Children with WD can have normal development at birth and then be asymptomatic ranging from months to years [12, 13]. Furthermore, not every WD patients present with KF (Kayser‐Fleischer) rings, hemolytic anemia, or low serum ceruloplasmin further complicating the timely diagnosis of the disease [7, 14]. For example, in a pediatric case series, only 38% of patients presented with KF rings at the time of diagnosis and only 25% presented with jaundice [15] in contrast, our patient presented with both jaundice and KF rings, highlight the diagnostic significant of this finding in atypical presentation.

Dependence solely on clinical and laboratory parameters (serum ceruloplasmin < 20 mg/dL, urinary copper excretion over 24 h > 100 μg/24 h) is inadequate for definitive diagnosis. Steindl (1997) [14] noted that 73%, 88%, and 55% of subjects with Wilson's disease demonstrated loss of ceruloplasmin, elevated urinary copper, and Kayser‐Fleischer rings present, respectively [14]. Likewise, hepatic copper content and ATP7B gene sequencing remain standard diagnostic tests but are not performed in many resource‐constrained settings [16]. The modified Leipzig score incorporates clinical, biochemical, and genetic information to improve diagnostic certainty for Wilson's disease. A cumulative score of 4 or greater supports the diagnosis of Wilson's disease, whilst imaging findings (ultrasound or Fibro Scan showing chronic liver inflammation) may bolster suspicion in equivocal cases [17, 18, 19]. The aforementioned diagnostic challenges relate specifically to Sudan, in that access to genetic testing and specialized imaging is often limited, thereby contributing to delays in diagnosis. For example, the low sensitivity of Kayser‐Fleischer rings (38% to 55%) and ceruloplasmin (65% in hepatic presentation) among cohorts in the literature [15, 16]. implies that clinicians in Sudan must maintain a high index of suspicion, particularly in patients with unexplained liver disease and/or neuropsychiatric symptoms.

Efforts to bridge the knowledge gap of the etiology of Wilson's disease (WD) in Sudan still necessitate prospective studies to characterize local genetic variants of ATP7B, environmental modifiers (dietary copper intake), and context‐specific diagnostic approaches and protocols. Addressing access to genetic testing and raising awareness of the heterogeneous presentations of WD will also be essential to reducing morbidity and mortality in this population group.

## 6 Conclusion

This case report highlights the phenomenon of Wilson disease (WD) in children in Africa (a population in which the diagnosis is commonly missed due to the lack of knowledge and diagnostic resources). The patient was a 13‐year‐old Sudanese male with consanguineous parents who had been suffering from hepatic (ascites, hypoalbuminemia) and progressive neuropsychiatric widely recognized (spasticity and seizures) conditions and had additional classic findings such as Kayser‐Fleischer rings and basal ganglia (basal ganglia) MRI changes (“giant panda sign”). The patient fulfilled the Leipzig criteria (score 7/10) and was commenced on chelation therapy; however, the swift degeneration of his neurological status, aggravated by the inability to access second‐line therapies (trientine), brought systemic issues in low‐resource settings to the forefront.

WD's phenotypic variability and reliance on specialized diagnostics (genetic testing, ceruloplasmin assays) pose significant challenges in regions like Sudan, where such tools are scarce. This case reinforces the need to consider WD in African children with unexplained liver dysfunction or extrapyramidal neurological symptoms, even in the absence of classic biochemical markers. Furthermore, it calls for urgent interventions to improve access to affordable diagnostics, essential medications, and multidisciplinary care in underserved regions.

## 7 Limitation

- The scarcity of Wilson Disease in Sudan may limit the generalizability of findings to other populations.
- The case report is based on a single patient's experience, which limits the potential to draw conclusions about the presentation and management of the disease.
- Some diagnostic tests (ceruloplasmin and 24‐h urinary copper) were not able to be performed due to a lack of resources, which may have impacted diagnostic accuracy and comprehensive diagnosis.
- The war in Sudan and subsequent medication shortages introduce environmental factors that could potentially affect the clinical course and outcomes for the patient and limit the ability to separate the effects of the disease from its treatment.
- Difficulty in long‐term monitoring of neurological and hepatic outcomes due to logistical barriers and the need for international referral for liver transplantation.

## 8 Recommendation

- Further study of wilson disease in sudan and other under‐represented areas.
- Larger studies to better characterize the disease and its presentation, progression, and response to treatment.
- Develop better healthcare infrastructure and access to diagnostic tools in low‐resource areas.
- Establish local registries for wilson disease and establish area‐specific management guidelines.

## Author Contributions

**Amjed Abdu Ali Mohammed:** writing – review and editing. **Anaheed Alzubair Mohammed HabeebAllah:** writing – original draft. **Sudad salahaldeen Hassan Mohammed:** writing – original draft, writing – review and editing. **Aprar Kamal Mohammed Ali Almamoon:** writing – original draft. **Mehad Mortada BadrAlden Ahmed:** writing – original draft. **Arafa Mubarak Abotalib Aref:** writing – original draft. **Fatima Bashir Omer Bashir:** investigation. **Tarig Mohamed Nourallah Altegani:** writing – review and editing. **Ahmed Alshafei Elmahi Ahmed:** writing – original draft, writing – review and editing.

## Funding

The authors have nothing to report.

## Ethics Statement

This study is exempt from ethical approval in our hospital.

## Consent

Written informed consent was obtained from the legal guardians of the patient for publication of this case report. The guardians acknowledged the report's public availability and confirmed that all identifiable information has been removed to protect the patients' privacy. Ethical approval for publication was granted by the Ethics Committee of Atbara Teaching Hospital, Sudan.

## Conflicts of Interest

The authors declare no conflicts of interest.

## Data Availability

The data that support the findings of this study are available on request from the corresponding author. The data are not publicly available due to privacy or ethical restrictions.
